# Omega-3 Fatty Acids and Cardiovascular Disease: Summary of the 2016 Agency of Healthcare Research and Quality Evidence Review

**DOI:** 10.3390/nu9080865

**Published:** 2017-08-11

**Authors:** Ethan M. Balk, Alice H. Lichtenstein

**Affiliations:** 1 Center for Evidence Synthesis in Health, Brown University School of Public Health, Providence, RI 02912, USA; ethan_balk@brown.edu; 2 Jean Mayer USDA Human Nutrition Research Center on Aging, Tufts University, Boston, MA 02111, USA

**Keywords:** omega-3 fatty acids, alpha-linolenic acid, eicosapentaenoic acid, docosahexaenoic acid, marine oil, cardiovascular disease, blood pressure, high density lipoprotein, low density lipoprotein cholesterol, triglyceride, systematic review, meta-analysis

## Abstract

We summarize the 2016 update of the 2004 Agency of Healthcare Research and Quality′s evidence review of omega-3 fatty acids and cardiovascular disease (CVD). The overall findings for the effects of marine oil supplements on intermediate CVD outcomes remain largely unchanged. There is high strength of evidence, based on numerous trials, of no significant effects of marine oils on systolic or diastolic blood pressures, but there are small, yet statistically significant increases in high density lipoprotein and low density lipoprotein cholesterol concentrations. The clinical significance of these small changes, particularly in combination, is unclear. The strongest effect of marine oils is on triglyceride concentrations. Across studies, this effect was dose-dependent and related to studies′ mean baseline triglyceride concentration. In observational studies, there is low strength of evidence that increased marine oil intake lowers ischemic stroke risk. Among randomized controlled trials and observational studies, there is evidence of variable strength of no association with increased marine oil intake and lower CVD event risk. Evidence regarding alpha-linolenic acid intake is sparser. There is moderate strength of evidence of no effect on blood pressure or lipoprotein concentrations and low strength of evidence of no association with coronary heart disease, atrial fibrillation and congestive heart failure.

## 1. Introduction

The relationship between high fish consumption and low cardiovascular mortality among Greenland Inuit was first reported in the late 1970s. Subsequently, numerous observational and intervention studies of fish and omega-3 fatty acids (*n*-3 FAs) intake have reported similar findings in many countries. The majority of the intervention trials have centered on cardiovascular disease (CVD) risk factors and intermediate markers. However, the beneficial effects on CVD risk factors and markers have not always been consistent with studies evaluating clinical CVD outcomes. Hence, the value of *n*-3 FA to decrease cardiovascular mortality and improve risk factors remains controversial.

The *n*-3 FAs are a group of long-chain and very-long-chain polyunsaturated fatty acids. The major *n*-3 FAs that are present in food are alpha-linolenic acid (ALA), occurring primarily in plants, and eicosapentaenoic acid (EPA) and docosahexaenoic acid (DHA), occurring primarily in marine life. Other *n*-3 FAs, including stearidonic acid (SDA) and docosapentaenoic acid (DPA), are present in very low amounts in the diet. The major dietary sources of ALA are soybean and canola oils, some nuts and flax seed. The major sources of EPA and DHA are oily fish and other marine life. Common dietary supplements of ALA are flax seed oil and some nut-derived oils. Common dietary supplements of EPA and DHA are fish oil, krill oil, and algae oil. There are no major commonly consumed sources of dietary SDA and DPA. However, SDA is relatively high in hemp oil and echium seed oil.

The term *n*-3 FAs is used to refer to a group of polyunsaturated fatty acids whose first double bond involves the third carbon counting from the methyl end of the fatty acid acyl chain. In contrast, the term omega-6 fatty acids (*n*-6 FA) is used to refer to a group of polyunsaturated fatty acids whose first double bond involves the sixth carbon counting from the methyl end of the fatty acid acyl chain. Both *n*-3 FAs and *n*-6 FAs are substrates for the synthesis of eicosanoids, a subcategory of oxylipins. As a group of bioactive molecules, they are signaling factors that affect a wide range of physiological systems. Depending on the substrate, eicosanoids can promote or inhibit immune responses, act as endocrine agents or have a broad range of other functions. The metabolic products of *n*-3 FAs and *n*-6 FAs tend to result in different and frequently opposite physiological effects. Metabolic products of *n*-3 FAs tend to be anti-inflammatory. In addition to being substrates for eicosanoid synthesis, *n*-3 FAs also serve as structural components of cell membranes, higher levels resulting in increased fluidity.

In 2002, the Institute of Medicine concluded that the evidence was inadequate to establish a Recommended Dietary Allowance for *n*-3 FAs. Instead, for healthy adults, they established an Adequate Intake for ALA of 1.1 g per day for females and 1.6 g per day for males [1,2]. On the basis of data for CVD and stroke, they further established an Acceptable Macronutrient Distribution Range for ALA of 0.6 to 1.2 percent of energy, with approximately 10 percent of this range contributed by EPA and/or DHA. To get an adequate *n*-3 FA intake, the 2015–2020 Dietary Guidelines for Americans recommends two fish meals, preferably oily fish, per week. This is consistent with prior editions of Dietary Guidelines for Americans and the American Heart Association′s Diet and Lifestyle Recommendations [3,4]. While the intake of ALA in the U.S. is generally adequate, intakes of EPA and DHA tend to be low. Despite consistent recommendations to increase fish intake, from 1999–2000 to 2011–2012 estimated fish intake has only increased from 1.12 to 1.33 servings per week [5].

In 2004, evidence reviews of *n*-3 FA and CVD and CVD risk factors commissioned by the Agency of Healthcare Research and Quality (AHRQ) were published [6,7,8,9,10]. Since then, the evidence for a relationship between *n*-3 FA and CVD has continued to be inconsistent. In the past decade, there have been numerous secular trends that may have had an impact upon the potential effects of *n*-3 FA dietary intake and supplementation on CVD risk factors and outcomes. These include higher diagnosis rates of and pharmacologic treatment for CVD risk factors (e.g., statins, anti-hypertensive agents, and low dose aspirin), resulting in lower cardiovascular event rates. Smoking rates have also fallen [11], although obesity rates have remained stable [12]. These trends could lower the potential population level benefit of *n*-3 FAs because of a lower underlying risk, making comparisons with older studies somewhat tenuous.

For these reasons, the AHRQ commissioned an update of the earlier review on *n*-3 FA and CVD [13]. The updated review focused on clinically relevant CVD risk factors (lipoproteins and blood pressure (BP)) and CVD events. In addition, due to concerns about the accuracy of dietary *n*-3 FA intake estimates, the updated review added evaluations of associations between measures of nutrient biomarkers and clinical outcomes. The biomarkers of *n*-3 FA intake include fatty acid profiles of adipose tissue, erythrocytes, plasma, and plasma phospholipids, reflecting not only current intake but subsequent metabolism [14,15,16]. The results of the updated review are summarized below.

## 2. Materials and Methods

**Figure 1:**
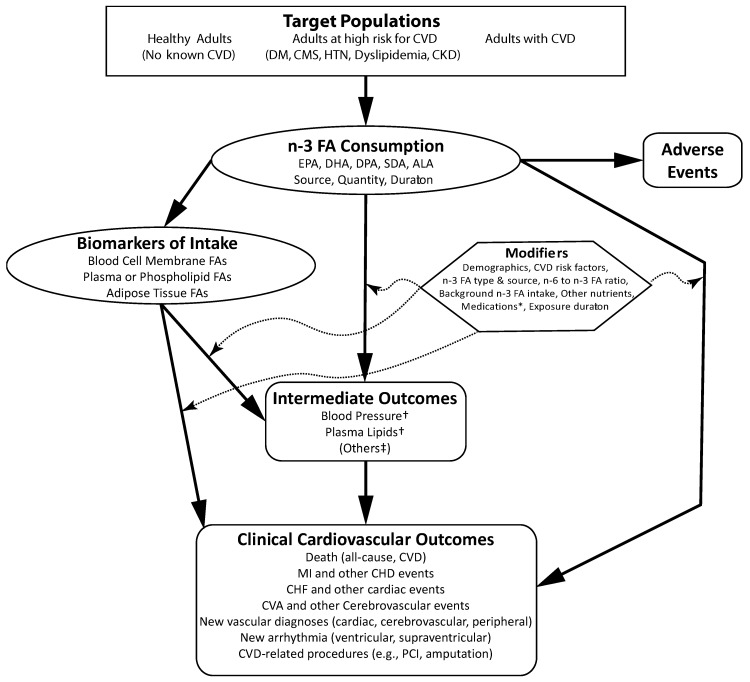
Analytic framework for omega-3 fatty acid exposure and cardiovascular disease.This framework concerns the effect of omega−3 fatty acid (*n*-3 FA) exposure (as a supplement or from food sources) on cardiovascular disease (CVD) events and risk factors. Populations of interest are noted in the top rectangle, exposure in the oval, outcomes in the rounded rectangles, and effect modifiers in the hexagon. * Specifically, cardiovascular medications, statins, anti-hypertensives, diabetes medications, hormone replacement regimens. ^†^ Systolic blood pressure, diastolic blood pressure, mean arterial pressure, high density lipoprotein cholesterol (HDL-c), low density lipoprotein cholesterol (LDL-c), total/HDL-C ratio, LDL-C /HDL-C ratio, triglycerides. ^‡^ Many other intermediate outcomes are likely in the causal pathway between n-3 FA intake and CVD outcomes, but only blood pressure and plasma lipids were included in the review. Other Abbreviations: ALA = alpha linolenic acid, CHD = coronary heart disease, CHF = congestive heart failure, CKD = non-dialysis-dependent chronic kidney disease, CMS = cardiometabolic syndrome, CVA = cerebrovascular accident (stroke), DHA = docosahexaenoic acid, DM = diabetes mellitus, DPA = docosapentaenoic acid, EPA = eicosapentaenoic acid, FA = fatty acid, HTN = hypertension, MI = myocardial infarction, *n*-6 = omega−6, PCI = percutaneous coronary intervention, SDA = stearidonic acid.

Standard systematic review methodology was employed to address three Key Questions on: (1) the efficacy or association of *n*-3 FA and CVD outcomes and risk factors; (2) differences in efficacy or association by patient characteristics, confounders, diet, and other factors on these outcomes and risk factors; and (3) adverse event data (Table 1). The Key Questions are summarized graphically in an Analytic Framework mapping linkages among populations of interest, exposures, modifying factors, and outcomes of interest (Figure 1). For each topic, the strength of evidence was rated as high, moderate, low, or insufficient, based on the number of studies, their limitations, consistency, precision, and other factors. Details about the study eligibility criteria and other methodology can be found in the full report [13].

## 3. Results

In total, 147 articles met eligibility criteria, representing 61 randomized controlled trials (RCT, in 82 articles) and 37 longitudinal observational studies (in 65 articles). Across studies, there were few risk of bias concerns. The RCTs of clinical outcomes were almost all conducted in populations at increased risk of CVD, largely related to dyslipidemia, or with CVD. The RCTs that reported intermediate outcomes (BP and lipoproteins) were conducted in generally healthy, at-risk, and CVD populations. The observational studies, in contrast, were almost all conducted in general (unrestricted by CVD or risk factors) or healthy populations.

### 3.1. Key Question 1: Efficacy or Association of n-3 FA and CVD Outcomes or Risk Factors

Findings of effects or associations of increased *n*-3 FA intake on CVD outcomes or risk factors are summarized in Table 2. Findings of no effect or association are summarized in Table 3. Details about study results and summaries across studies can be found in the full report [13].

#### 3.1.1. Total *n*-3 FA

Overall, there is insufficient evidence regarding the effect of or association between total n-3 FA (combined ALA and marine oils) and most clinical and intermediate outcomes. There is low strength of evidence of no association between total *n*-3 FA intake and stroke death, and total (fatal and nonfatal) myocardial infarct, based on longitudinal observational studies of dietary intake.

#### 3.1.2. Marine Oils

There is high strength of evidence of that marine oils (primarily EPA and DHA) statistically significantly lower triglyceride concentrations—possibly with greater effects with higher doses and in people with higher baseline triglyceride concentrations—and they statistically significantly raise high density lipoprotein cholesterol (HDL-C) and low density lipoprotein cholesterol (LDL-C) concentrations by similar amounts (2.0 and 0.9 mg/dL, respectively). There is also high strength of evidence that marine oils significantly lower the total cholesterol (TC)/HDL-C ratio (by −0.17). There is low strength of evidence that marine oils significantly lower risk of ischemic stroke (effect size per g/day = 0.51).

There is a high strength of evidence of no effect of marine oils on risk of major adverse cardiovascular events, all-cause death, sudden cardiac death, revascularization, and blood pressure (BP); moderate strength of evidence of no effect of marine oils on risk of atrial fibrillation; and low strength of evidence of no effect of marine oils on risk of CVD death, coronary heart disease (CHD) death, total CHD, myocardial infarction, angina pectoris, congestive heart failure, total stroke, and hemorrhagic stroke. There is insufficient evidence for other outcomes.

#### 3.1.3. Specific Marine Oils

There is insufficient evidence regarding the effect of or association between oils high in EPA, DHA, or DPA (each marine oil individually) and most CVD clinical and intermediate outcomes. There is low strength of evidence of no association between EPA intake and CHD and between EPA biomarkers and atrial fibrillation. There is moderate strength of evidence of no effect of purified DHA supplementation on BP or LDL-C concentrations, and low strength of evidence of no association between DHA intake and incident CHD. There is low strength of evidence of no association between DPA biomarker levels and risk of atrial fibrillation.

There is insufficient evidence regarding effect of or association between SDA and CVD clinical and intermediate outcomes.

There is moderate strength of evidence of no significant effect of ALA intake on BP, or LDL-C, HDL-C, and triglyceride concentrations. There is low strength of evidence of no association between ALA intake or biomarker level and CHD or CHD death, atrial fibrillation, and congestive heart failure, each based on observational studies. There is insufficient evidence regarding other outcomes.

#### 3.1.4. Sub-Questions

##### 1. People with No Known CVD, At Increased Risk for CVD, and With Known CVD.

Almost all studies reporting CVD intermediate outcomes included study participants based on BP or lipoprotein concentrations; i.e., at increased risk for CVD, but no known CVD. Most observational studies evaluated general population registries or other large databases (for primary prevention). In contrast, RCTs with CVD event outcomes were conducted mostly in people with known history of CVD (for secondary prevention).

Based on the applicability of the different studies, in the population without known CVD, there is observational evidence of no association for major adverse cardiovascular events, CVD death, total stroke death, incident CHD, total stroke, ischemic stroke, hemorrhagic stroke, atrial fibrillation, and congestive heart failure. There is strong RCT evidence of no effect for BP (systolic and diastolic), mean arterial pressure, LDL-C and HDL-C concentrations, and strong RCT evidence for a significant effect on lowering triglyceride concentrations.

In people at increased risk for CVD, there is strong RCT evidence for no effect on major adverse cardiovascular events, all-cause death, BP (systolic and diastolic), LDL-C and HDL-C concentrations, TC/HDL-C ratio, and LDL-C/HDL-C ratio, and strong RCT evidence for a significant effect for lowering triglyceride concentrations.

In people with known CVD, there is RCT evidence of no effect for major adverse cardiovascular, CHD death, all-cause death, myocardial infarction, revascularization, total stroke, sudden cardiac death, atrial fibrillation, and congestive heart failure. There is strong RCT evidence of no effect on BP (systolic and diastolic) and LDL-C concentrations, and strong RCT evidence of a protective effect for HDL-C and triglyceride concentrations.

##### 2. Relative Effect of Different *n*-3 FAs.

Based on studies that directly compared different n-3 FAs, there is low strength of evidence of no difference between EPA, DHA, and combined EPA+DHA. There is low strength of evidence of greater efficacy of marine oils over ALA.

##### 3. Ordering of *n*-3 FAs by Strength of Effect.

Based on the summary effect sizes of meta-analyzed RCTs, marine oils had no statistically significant effect on CVD outcomes. The order of effect sizes (ignoring lack of statistical significance) of CVD outcomes with sufficient data to allow meta-analysis, was myocardial infarction, CVD death, major adverse cardiovascular events, all-cause death, total stroke, and sudden cardiac death.

### 3.2. Key Question 2: n-3 FA Variables and Modifiers

#### Sub-Questions

##### 1. Subpopulations

There was insufficient evidence to assess the efficacy or association of *n*-3 FA in preventing CVD outcomes and with CVD risk factors in subgroups based on race/ethnicity and whether women were pre- or postmenopausal. Five studies (mostly observational) found no significant differences in association based on age, with cutoffs for subgroups ranging between 60 and 70 years of age. Two studies found no interaction with age as a continuous variable. One RCT found a significant difference in favor of women, two observational studies found a significant difference in favor of men, and nine studies (a mix of RCTs and observational) found no difference between men and women.

##### 2. Confounders or Interacting Factors

There was evidence of no interactions with body mass index, hypertension status, diabetes status, and baseline TC/HDL-C ratio. There was inconsistent evidence for the following potential confounders or interacting factors: triglyceride concentrations, statin use, B-vitamin use, and baseline LDL-C concentrations. There was insufficient evidence to assess the following potential confounders or interacting factors: beta-blocker use, baseline HDL-C concentrations, insulin glargine use, nitrate use, digoxin use, diuretic use, estimated glomerular filtration rate, angiotensin-converting enzyme inhibitor use, anticoagulant use, total cholesterol concentrations, or use of fish oil supplements.

##### 3–6. Different Ratios of *n*-3 FA Components, Different *n*-3 FA Sources, and *n*-6 FA to *n*-3 FA Ratio

There was insufficient information across studies to evaluate different ratios of *n*-3 FA components (e.g., EPA-to-DHA ratio) or to compare different ratios. Also, studies neither fully reported on *n*-3 FA source (e.g., soybean oil, canola oil) nor compared the different sources; therefore, there was insufficient evidence regarding differential effects based on source. No RCTs or observational studies directly evaluated *n*-6 FA to *n*-3 FA intake concentrations, and no differences across studies by this ratio was evident.

##### 7. Threshold or Dose–Response Relationship

Among RCTs, for all clinical CVD outcomes, there is insufficient evidence regarding a dose–response relationship within or between RCTs. For BP, LDL-C and HDL-C concentrations, RCTs do not find significant differences in effect by marine oil dose either within or between RCTs. RCTs comparing marine oil doses mostly found no significant differences between higher and lower dose marine oils. However, a possible pattern could be discerned such that higher doses (3.4 or 4 g/day) reduced triglyceride concentrations by at least 30 mg/dL more than lower doses (1 to 2 g/day). By meta-regression, each increase of EPA+DHA dose by 1 g/day was associated with a greater net change triglyceride concentrations of −5.9 mg/dL (95% CI −9.9 to−2.0; *P* = 0.003); no inflection point was found above which the association plateaued. Meta-regressions of observational studies yielded the following conclusions. For all-cause death, there may be a ceiling effect at about 0.2 g/day, such that increasing marine oil intake up to this level may be associated with lower all-cause death, but increasing intake above this level may not be associated with further decreased risk. For total stroke, ischemic stroke, and congestive heart failure, at lower ranges of intake, there were statistically significant associations between higher marine oil intake level and lower risk of outcome, in contrast to associations found at higher ranges of intake. However, the associations at lower and higher doses were not statistically significantly different from each other. For ischemic stroke, associations between higher doses and risk of stroke were stronger and statistically significant across lower doses than at higher doses (with thresholds between lower and higher doses from 0.1 and 0.4 g/day) and the differences in associations between lower and higher doses were statistically significant. Any dose inflection point that may exist is likely to be beyond the range of testable thresholds (i.e., >0.4 g/day), based on available evidence. Similarly, for congestive heart failure, significant associations were found at lower doses, in contrast to at higher doses, with thresholds ranging from 0.1 to 0.5 g/day, and the differences were statistically significant at most thresholds. Any dose inflection point that may exist is likely to be beyond the range of testable thresholds (i.e., >0.5 g/day). For CVD death, CHD death, total CHD, and hemorrhagic stroke, there were no apparent differences in association between marine oil intake dose and outcome at lower or higher dose ranges. For CHD death and CHD, there were no apparent differences in association between ALA intake dose and outcome at lower or higher dose ranges.

##### 8. Duration of Intervention or Exposure

None of the meta-regressions identified a significant interaction for follow-up time. No difference in effect was identified within studies at different durations of intervention. Observational studies did not evaluate differences in duration of exposure.

##### 9. Effect of Baseline *n*-FA Status

The very few studies that investigated potential differential effects associated with baseline fish or *n*-3 FA intake found no significant differences.

### 3.3. Adverse Events

#### Sub-Questions

##### 1. Adverse Events across All Studies

No serious or severe adverse events were related to *n*-3 FA intake (supplementation). Most reported adverse events were mild and gastrointestinal in nature. However, two of 25 RCTs reported statistically significant differences in adverse events between *n*-3 FA supplements and placebo.

##### 2. Adverse Events among People with CVD or Diabetes

Among 10 RCTs of patients with CVD (9 with marine oil, 1 with total *n*-3 FA, 2 with ALA), either no adverse events or no significant difference between *n*-3 FA and placebo were reported. A single study reported adverse events from an RCT of people with diabetes, finding no significant differences in serious or non-serious adverse events between marine oil and placebo.

## 4. Discussion

The overall findings for the effects of marine oil supplements on intermediate CVD outcomes remain largely unchanged since a similar review in 2004 [7,13]. In summary, there is high strength of evidence, based on numerous trials, of no significant effects of marine oils (0.3–6 g/day) on systolic or diastolic BP, but small, yet statistically significant increases in HDL-C (0.9 mg/dL) and LDL-C (2.0 mg/dL) concentrations. However, the clinical significance of these small changes in both HDL-C and LDL-C concentrations on CVD outcomes, particularly in combination, is unclear. For both lipid outcomes, no differences in effect across studies were found by marine oil dose, follow-up duration, or population. The strongest effect of marine oils (0.3–6 g/day) was found on triglyceride concentrations. Across studies, this effect was dose-dependent and also dependent on the studies′ mean baseline triglyceride concentrations. Recent genetic evidence from a wide range of investigations, including mutational analyses, genome-wide associations and Mendelian randomization, has linked triglycerides and triglyceride-rich lipoprotein particles in the causal pathway for CVD, possibly through promotion of low-grade inflammation [17,18].

In observational studies, there is a low strength of evidence that increased marine oil intake lowers the risk of ischemic stroke, but among both RCTs and observational studies there is evidence of variable strength of no association of increased marine oil intake with lower risks of a range of CVD events. Evidence regarding ALA intake is sparser. There is moderate strength of evidence of no effect of ALA on BP or lipoprotein concentrations, and low strength of evidence of no association with coronary heart disease, atrial fibrillation, and congestive heart failure.

The potential intake threshold-effects of *n*-3 FA on CVD events (the maximum dose above which no further benefit is attained) could not be determined from the RCTs; meta-analyses of observational studies found variable evidence of possible threshold effects. Most notably, intakes of EPA and DHA greater than 0.6 g/day do not provide additional benefit to lower risk of ischemic stroke than lower doses. Comparative differences in effects or associations of increased *n*-3 FA intake in different populations based on CVD risk, a question of particular interest, could not be adequately addressed because few RCTs were conducted in healthy populations (with normal CVD risk) and few observational studies were conducted in at-risk or CVD populations.

Of interest, the current National Institute for Health and Care Excellence (NICE) recommendations for CVD prevention concluded that the evidence does not support the use of omega-3 fatty acid supplements for people who are being treated for primary prevention or secondary prevention, and people with chronic kidney disease, type 1 diabetes, or type 2 diabetes [19].

## 5. Limitations

Overall, both RCTs and observational studies generally had few risk of bias concerns. However, as noted, the RCTs were mostly applicable to people with elevated BP or lipoprotein concentrations, without known CVD. In contrast, for clinical CVD outcomes, all but one of the RCTs was conducted in either high-risk individuals or people with existing CVD, but most observational studies were conducted in generally healthy populations. Furthermore, the doses of marine oil supplements in RCTs were often much higher than the highest intake reported for observational studies. Studies generally failed to account for differences in background diet or *n*-3 FA intake and did not fully characterize the *n*-3 FA under investigation.

While this report represents a complete systematic review, it does not encompass all trials or longitudinal observational studies that report on CVD and intermediate outcomes. Due to time and resource constraints, this review included only the largest RCTs of CVD risk factors and the largest observational studies. Smaller studies may have yielded more complete or conflicting findings.

## 6. Conclusions

In brief, there is high strength of evidence that marine oils have small effects on LDL-C and HDL-C concentrations, and a large, dose-dependent effect on triglyceride concentrations. In contrast, there is moderate strength of evidence that ALA has no significant effect on lipoprotein concentrations. Neither marine oils nor ALA have a significant effect on systolic or diastolic BP. There is moderate strength of evidence that marine oil supplementation lowers risk of major adverse cardiovascular events and CVD death, and low strength of evidence that higher marine oil intake is associated with lower risk of coronary heart disease and congestive heart failure. However, there is variable strength of evidence of no significant effect or association of marine oil intake and numerous different CVD outcomes.

The generalizability of specific findings to all populations is somewhat limited because studies tended to restrict their eligibility criteria. RCTs evaluating clinical outcomes included only people with known CVD while observational studies evaluated only databases of generally health populations (without known CVD). Furthermore, while there were few risk of bias concerns across the studies, very few studies fully characterized the *n*-3 FAs under investigation or attempted to account for differential effects in different populations or based on people′s background diet or other characteristics. Also, few studies directly compared different *n*-3 FA components, ratios, doses, or duration of intake. Therefore, there was no or insufficient evidence to address most of the review′s key questions.

## Figures and Tables

**Table 1:** Key Questions.

Key Question	Question Text
1	What is the efficacy or association of *n*-3 FA (EPA, DHA, EPA+DHA, DPA, SDA, ALA, or total *n*-3 FA) exposures in reducing CVD outcomes (incident CVD events, including all-cause death, CVD death, nonfatal CVD events, new diagnosis of CVD, peripheral vascular disease, CHF, major arrhythmias, and hypertension diagnosis) and specific CVD risk factors (BP, key plasma lipids)?
1.1	What is the efficacy or association of *n*-3 FA in preventing CVD outcomes in people • Without known CVD (primary prevention) • At high risk for CVD (primary prevention), and • With known CVD (secondary prevention)?
1.2	What is the relative efficacy of different *n*-3 FA on CVD outcomes and risk factors?
1.3	Can the CVD outcomes be ordered by strength of intervention effect of *n*-3 FA?
2	*n*-3 FA variables and modifiers:
2.1	How does the efficacy or association of *n*-3 FA in preventing CVD outcomes and with CVD risk factors differ in subpopulations, including men, premenopausal women, postmenopausal women, and different age or race/ethnicity groups?
2.2	What are the effects of potential confounders or interacting factors—such as plasma lipids, body mass index, BP, diabetes, kidney disease, other nutrients or supplements, and drugs (e.g., statins, aspirin, diabetes drugs, hormone replacement therapy)?
2.3	What is the efficacy or association of different ratios of *n*-3 FA components in dietary supplements or biomarkers on CVD outcomes and risk factors?
2.4	How does the efficacy or association of *n*-3 FA on CVD outcomes and risk factors differ by ratios of different *n*-3 FA—DHA, EPA, and ALA, or other *n*-3 FA?
2.5	How does the efficacy or association of *n*-3 FA on CVD outcomes and risk factors differ by source (e.g., fish and seafood, common plant oils (e.g., soybean, canola), fish oil supplements, fungal-algal supplements, flaxseed oil supplements)?
2.6	How does the ratio of *n*-6 FA to *n*-3 FA intakes or biomarker concentrations affect the efficacy or association of *n*-3 FA on CVD outcomes and risk factors?
2.7	Is there a threshold or dose–response relationship between *n*-3 FA exposures and CVD outcomes and risk factors? Does the study type affect these relationships?
2.8	How does the duration of intervention or exposure influence the effect of *n*-3 FA on CVD outcomes and risk factors?
2.9	What is the effect of baseline *n*-3 FA status (intake or biomarkers) on the efficacy of *n*-3 FA intake or supplementation on CVD outcomes and risk factors?
3	Adverse events:
3.1	What adverse effects are related to *n*-3 FA intake (in studies of CVD outcomes and risk factors)?
3.2	What adverse events are reported specifically among people with CVD or diabetes (in studies of CVD outcomes and risk factors)?

Abbreviations: ALA = alpha-linolenic acid, BP = blood pressure, CHF = congestive heart failure, CVD = cardiovascular disease, DHA = docosahexaenoic acid, DPA = docosapentaenoic acid, EPA = eicosapentaenoic acid, *n*-3 FA = omega-3 fatty acid(s), *n*-6 FA = omega-6 fatty acid(s), SDA = stearidonic acid.

**Table 2:** Main findings of high, moderate, or low strength of evidence of significant effects or associations between omega-3 fatty acids and outcomes.

Effect or Association	Strength of Evidence	Finding	Study Types	Effect Sizes
Higher *n*-3 FA intake or biomarker levels with lower CVD risks or events	High	Marine Oil * Supplementation (Or Increased Intake) Raises HDL-C	RCTs (of mostly supplements)	Summary net change in HDL-C: 0.9 mg/dL (95% CI 0.2, 1.6)
	High	Marine oil supplementation (or increased intake) lowers Tg	RCTs (of mostly supplements)	Summary net change in Tg: −24 mg/dL (95% CI −31, −18)
	High	Marine oil supplementation (or increased intake) lowers TC/HDL-C ratio	RCTs (of mostly supplements)	Summary net change in TC/ HDL-C ratio: −0.17 (95% CI −0.26, −0.09)
	Low	Marine oil increased intake lowers risk of ischemic stroke	Observational studies (of total dietary intake)	By metaregression: 0.51 (95% CI 0.29, 0.89) per g/day
Higher *n*-3 FA intake or biomarker levels with higher CVD risk	High	Marine Oil Supplementation (Or Increased Intake) Raises LDL-C	RCTs (of mostly supplements)	Summary net change in LDL-C: 2.0 mg/dL (95% CI 0.4, 3.6)

* All statements about “marine oil” are based on all evidence of analyses of EPA+DHA+DPA, EPA+DHA, EPA, DHA, and DPA. Abbreviations: CHD = coronary heart disease (also known as coronary artery disease), CHF = congestive heart failure, CI = confidence interval, CVD = cardiovascular disease, DHA = docosahexaenoic acid, DPA = docosapentaenoic acid, EPA = eicosapentaenoic acid, HDL-C = high density lipoprotein cholesterol, HR = hazard ratio, LDL-C = low density lipoprotein cholesterol, *n*-3 FA = omega-3 fatty acids, RCT = randomized controlled trial, TC = total cholesterol, Tg = triglycerides.

**Table 3:** Main findings of high, moderate, or low strength of evidence of no significant effects or associations between omega-3 fatty acids and outcomes.

Strength of Evidence	Omega-3 Fatty Acid and Outcome	Study Types	Summary Effect Sizes
High	Marine oil* supplementation (or increased intake) and MACE	RCTs (of mostly supplements), supported by observational studies (of total dietary intake)	RCTs: 0.96 (95% CI 0.91, 1.02)
High	Marine oil intake and all-cause death	RCTs (of mostly supplements) and observational studies (of total dietary intake)	RCTs: 0.97 (95% CI 0.92, 1.03). Observational studies: 0.62 (95% CI 0.31, 1.25) per g/day
High	Marine oil intake and SCD	RCTs (of mostly supplements), supported by observational studies (of total dietary intake)	RCTs: 1.04 (95% CI 0.92, 1.17)
High	Marine oil intake and coronary revascularization	RCTs (of mostly supplements), supported by observational studies (of total dietary intake)	Not significant, not meta-analyzed
High	Marine oil intake and systolic or diastolic blood pressure	RCTs (of mostly supplements)	RCTs: summary net change in systolic blood pressure: 0.1 mg/dL (95% CI −0.2, 0.4); summary net change in diastolic blood pressure: −0.2 mg/dL (95% CI −0.4, 0.5)
Moderate	Marine oil intake and atrial fibrillation	RCTs (of mostly supplements) and observational studies (of total dietary intake)	Not significant, not meta-analyzed. Observational studies were inconsistent.
Moderate	Purified DHA supplementation and systolic or diastolic blood pressure	RCTs (of supplements)	Not significant, not meta-analyzed
Moderate	Purified DHA supplementation and LDL-C	RCTs (of supplements)	Not significant, not meta-analyzed
Moderate	ALA intake and systolic or diastolic blood pressure	RCTs (of mostly supplements)	Not significant, not meta-analyzed
Moderate	ALA intake and LDL-C, HDL-C, and Tg	RCTs (of mostly supplements)	Not significant, not meta-analyzed
Low	Total *n*-3 FA intake and stroke death	Observational studies (of total dietary intake and biomarkers)	Not significant, not meta-analyzed
Low	Total *n*-3 FA intake and myocardial infarction	Observational studies (of total dietary intake)	Not significant, not meta-analyzed
Low	Marine oil intake and CVD death	RCTs (of mostly supplements) and observational studies (of total dietary intake)	RCTs: 0.92 (95% CI 0.82, 1.02). Observational studies: 0.88 (95% CI 0.82, 0.95) per g/day
Low	Marine oil intake and CHD death	RCTs (of mostly supplements) and observational studies (of total dietary intake)	RCTs imprecise. Observational studies: 1.09 (95% CI 0.76, 1.57) per g/day
Low	Marine oil intake and CHD	Observational studies (of total dietary intake and biomarkers)	Observational studies: 0.94 (95% CI 0.81, 1.10) per g/day
Low	Marine oil intake and myocardial infarction	RCTs (of mostly supplements)	RCTs: 0.88 (95% CI 0.77, 1.02)
Low	Marine oil intake and angina pectoris	RCTs (of mostly supplements)	Not significant, not meta-analyzed
Low	Marine oil intake and CHF	RCTs (of mostly supplements) and observational studies (of total dietary intake)	RCTs not significant, not meta-analyzed. Observational studies: 0.76 (95% CI 0.58, 1.00) per g/day
Low	Marine oil intake and total stroke (fatal and nonfatal ischemic and hemorrhagic stroke)	RCTs (of mostly supplements) and observational studies (of total dietary intake)	RCTs: 0.97 (95% CI 0.83, 1.13). Observational studies: 0.68 (95% CI 0.53, 0.87) per g/day
Low	Marine oil intake and hemorrhagic stroke	Observational studies (of total dietary intake)	Observational studies: 0.61 (95% CI 0.34, 1.11) per g/day
Low	EPA intake and CHD	Observational studies (of total dietary intake)	Not significant, not meta-analyzed
Low	EPA biomarkers and atrial fibrillation	Observational studies (of biomarkers)	Not significant, not meta-analyzed
Low	DHA intake and CHD	Observational studies (of total dietary intake and biomarkers)	Not significant, not meta-analyzed
Low	DPA biomarkers and atrial fibrillation	Observational studies (of biomarkers)	Not significant, not meta-analyzed
Low	ALA intake and CHD death	Observational studies (of total dietary intake), supported by RCT (of supplementation) and observational study (of biomarkers)	Observational studies: 0.94 (95% CI 0.85, 1.03) per g/day
Low	ALA intake and CHD	Observational studies (of total dietary intake)	Observational studies: 0.97 (95% CI 0.92, 1.03) per g/day
Low	ALA intake and atrial fibrillation	Observational studies (of total dietary intake and biomarkers)	Not significant, not meta-analyzed
Low	ALA intake and CHF	Observational studies (of total dietary intake and biomarkers), supported by RCT (of supplementation)	Not significant, not meta-analyzed

* All statements about “marine oil” are based on all evidence of analyses of EPA+DHA+DPA, EPA+DHA, EPA, DHA, and DPA. Abbreviations: ALA = alphalinolenic acid, CHD = coronary heart disease, CHF = congestive heart failure, CI = confidence interval, DHA = docosahexaenoic acid, DPA = docosapentaenoic acid, EPA = eicosapentaenoic acid, HDL-C = high density lipoprotein cholesterol, LDL-C = low density lipoprotein cholesterol, MACE = major adverse cardiovascular event (including cardiac and stroke events and death; variously defined by studies), *n*-3 FA = omega-3 fatty acids, RCT = randomized controlled trial, SCD = sudden cardiac death, Tg = triglycerides.

## Disclaimer

The authors of this manuscript are responsible for its content. Statements in the manuscript should not be construed as endorsement by the AHRQ or the U.S. Department of Health and Human Services. AHRQ retains a license to display, reproduce, and distribute the data and the report from which this manuscript was derived under the terms of the agency′s contract with the authors.
